# Intuitive and versatile bionic legs: a perspective on volitional control

**DOI:** 10.3389/fnbot.2024.1410760

**Published:** 2024-06-20

**Authors:** Matthias Voß, Anne D. Koelewijn, Philipp Beckerle

**Affiliations:** ^1^Chair of Autonomous Systems and Mechatronics, Department Electrical Engineering, Friedrich-Alexander-Universität Erlangen-Nürnberg, Erlangen, Germany; ^2^Department Artificial Intelligence in Biomedical Engineering, Friedrich-Alexander-Universität Erlangen-Nürnberg, Erlangen, Germany

**Keywords:** lower limb prostheses, bionic legs, voluntary control, electromyography, human-in-the-loop optimization

## Abstract

Active lower limb prostheses show large potential to offer energetic, balance, and versatility improvements to users when compared to passive and semi-active devices. Still, their control remains a major development challenge, with many different approaches existing. This perspective aims at illustrating a future leg prosthesis control approach to improve the everyday life of prosthesis users, while providing a research road map for getting there. Reviewing research on the needs and challenges faced by prosthesis users, we argue for the development of versatile control architectures for lower limb prosthetic devices that grant the wearer full volitional control at all times. To this end, existing control approaches for active lower limb prostheses are divided based on their consideration of volitional user input. The presented methods are discussed in regard to their suitability for universal everyday control involving user volition. Novel combinations of established methods are proposed. This involves the combination of feed-forward motor control signals with simulated feedback loops in prosthesis control, as well as online optimization techniques to individualize the system parameters. To provide more context, developments related to volitional control design are touched on.

## 1 Introduction

Research in active lower limb prostheses has recently received increasingly more attention. While passive prostheses only restore and return energy during the movement of the user and semi-active devices modulate this energy return by changing system dynamics, active prostheses are able to provide net positive energy to their wearer. Besides the energetic benefit and more possible movement applications, active leg prostheses have been shown to improve balance (Berry, 2006), functional performance, satisfaction and quality of life compared to passive devices (Burçak et al., 2021). Meanwhile, the design of device controllers that benefit users in locomotor tasks amounts to one of the main challenges in the development of active prostheses (Voloshina and Collins, 2020).

To maximize the potential benefits of active prosthesis hardware in the long term, wearers should be able and eager to use them as often as possible, which necessitates user satisfaction. In general, prosthesis usefulness is strongly associated with embodiment, which itself correlates with user satisfaction (Bekrater-Bodmann, 2021). More specifically, certain aspects to prosthesis usage satisfaction are directly affected by the control architecture in place. Versatility and intuitiveness in leg prostheses support independence, confidence and safety, which are key user desires (Manz et al., 2022). A lack of voluntary motor functionality can lead to user dissatisfaction (Christ et al., 2011). A recent systematic review identified users' ability to perform different movements as a factor for satisfaction with lower limb prostheses, amongst others (Baars et al., 2018). These relations call for control methods allowing users to modulate the prosthesis behavior according to certain movement tasks and environments or, more universally, at will. We refer to this approach as volitional control.

The most practical and technologically matured, noninvasive way to transfer control commands from the user to a prosthetic device is electromyography (EMG) (Zheng, 2019). People with lower limb amputations are able to volitionally use their residual muscles for EMG-based control (Huang and Huang, 2018, 2019). There are challenges to EMG control, like differing muscle activation profiles between individuals (Huang and Ferris, 2012), as well as varying abilities to volitionally create activation patterns, including unintended coactivation of antagonistic muscles (Huang and Huang, 2019). However, users are able to learn and improve their control capabilities over time (Alcaide-Aguirre et al., 2013; Fleming et al., 2018).

To date, the majority of lower-limb prostheses is autonomously controlled based on state prediction (Fleming et al., 2021), which constitutes a discrepancy between the potential benefit of intuitive volitional control to the users and the control strategies implemented in research and commercial prosthesics. Currently, there are no commercially available devices using EMG (Fleming et al., 2021; Ahkami et al., 2023) and even in research, only half of EMG leg prosthesis are volitionally controllable at most, as recent reviews show (Fleming et al., 2021; Cimolato et al., 2022; Ahkami et al., 2023). Besides numerous experiments involving pointing tasks or virtual lower limbs, only a few studies cover initial trials with volitional control during gait. When considering the enabling of gait the primary function of a leg prosthesis, the state of the art of volitionally controllable leg prostheses is deficient. Therefore, we argue for the development of a universally applicable volitional control architecture suitable for everyday use. For this purpose, this perspective offers an overview of existing control strategies and assesses their respective applicability. The combination of these control approaches is discussed to point out directions in future research in this field. Additionally, other potential methods to advance volitional control, namely restoration of proprioception and human-in-the-loop optimization, are examined.

## 2 Lower limb prosthesis control strategies

**Figure 1:**
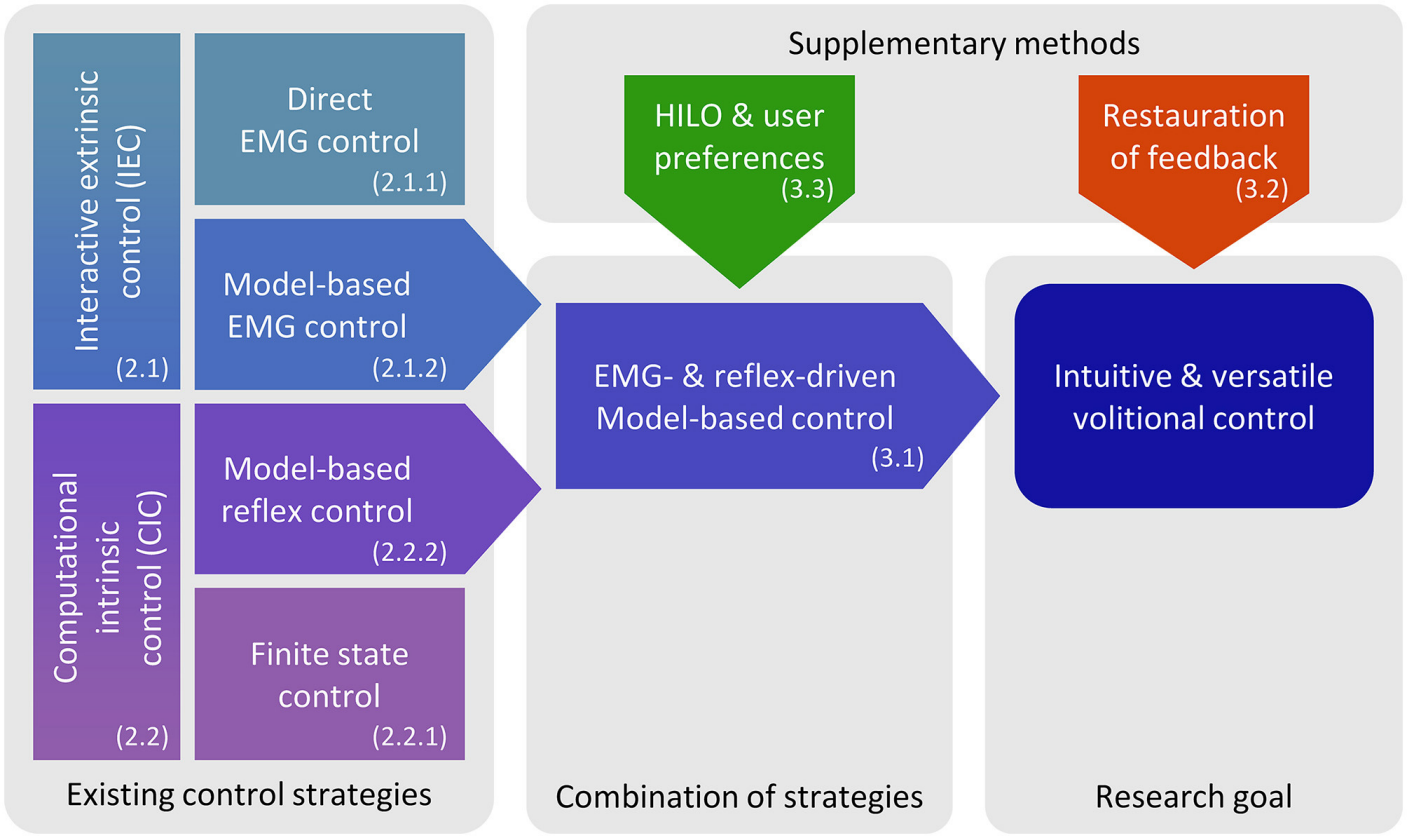
Schematic overview of control approaches and research roadmap. From each category of existing control strategies (IEC, CIC), one model-based approach is selected to be incorporated in a hybrid control architecture. Human-in-the-loop optimization (HILO) is meant to tailor the model to individual users, while the artificial restoration of device state feedback shall improve the user's control abilities.

This section summarizes existing control strategies for active lower limb prostheses and discusses their suitability for volitional control to derive the novel control architecture proposed in Section 3. As suggested by Martin et al. (2010), we divide the control approaches in two categories, depending on how they incorporate inputs from the user's nervous system. Interactive extrinsic control (IEC) assigns the prosthesis user immediate and continuous control of the device's behavior, with the commonly used interface being surface EMG from the muscles in the residual limb. In contrast to IEC, computational intrinsic control (CIC) does not receive direct volitional input by the user and instead determines prosthesis movement autonomously (Cimolato et al., 2022). Device-specific low-level controllers setting torque, position, or speed on the prosthetic hardware are not affected by this distinction and not discussed here. The presented categories and their proposed consideration in volitional prosthesis control are shown in Figure 1. The examples mentioned focus on implementations that enable gait, whereas non-weight-bearing tasks and virtual devices are not discussed.

### 2.1 Interactive extrinsic control (IEC)

Since IEC enables the user to continuously and directly modulate the prosthesis state, its inclusion in a volitional control architecture appears obvious. A general detriment to IEC is its constant reliance on user input. One can assume this increases cognitive user load compared to autonomously controlled devices, but this has yet to be quantified (Fleming et al., 2021). This potential downside applies to both IEC variants presented in the following.

#### 2.1.1 Direct EMG control

Direct EMG control uses mathematical functions to calculate a desired prosthesis state such as joint angle or torque from EMG input, with only a few papers discussing its application for gait. Huang et al. (2014, 2016) used EMG-proportional pressure in artificial pneumatic muscles to control an ankle prosthesis, which allowed level-ground walking under the provision of visual feedback. Other studies implemented direct control with an electromechanical knee prosthesis (Hoover et al., 2012; Dawley et al., 2013). Here, for level-ground walking and stair ascend, the stiffness and equilibrium point of impedance controllers were modulated via recorded EMG signals, producing a compliant volitional position control.

An advantage of direct control is its relatively low computational cost (Ahkami et al., 2023), especially for simple approaches like proportional control. Since the method does not inherently mimic any biological example, it might at times generate unnatural relations between muscle activity and prosthesis behavior (e.g., proportional joint torque), that are hard to learn for users. Though, to point out a clear tendency here, the number of conducted experiments with this approach is too small.

#### 2.1.2 Muscle-model-based EMG control

In muscle-model-based EMG prosthesis control, a simplified simulation of EMG-driven muscles acting on a joint is employed to calculate a resulting joint torque. Here, EMG amplitudes modulated the spring stiffness, damper coefficient, and a proportional force to calculate the desired knee torque. This enabled level-ground walking by an able-bodied individual wearing a prosthesis adapter. Shah et al. (2022) used two Hill-type-muscles driven by gastrocnemius and tibialis anterior EMG recordings to control an ankle prosthesis for a symmetrical balancing task.

In model-based EMG control, the natural joint behavior depending on muscle activity is emulated and driven by a volitional input. Compared to direct EMG control, a downside of this approach is a rather complex model. This likely leads to increased computational demand and higher numbers of model parameters to be determined.

### 2.2 Computational intrinsic control (CIC)

The lack of immediate user authority over the device offered by CIC seems to rule this control approach out for volitional control at first glance. We still discuss CIC variants to get a comprehensive view on existing control approaches and evaluate their suitability to maybe be partially incorporated in a volitional approach.

#### 2.2.1 Finite state controllers

Finite state controllers choose between a finite number of operation modes, target trajectories, set points or other system parameters to tackle a given task. To make the system adaptable to changing walking speeds, gait modes or environments, a recognition of gait phases or user intend is required. Besides sensors for non-biosignals like inertial or force sensors, EMG can also be incorporated for this classification task (Cimolato et al., 2022). With modern machine learning approaches, low single-digit percentage classification errors have been achieved (Voloshina and Collins, 2020).

For prosthesis users, classification errors are hard to comprehend and hard to compensate for, since the mapping of EMG input to the prosthesis behavior is a complex black box (Fleming et al., 2021). Depending on their type and timing, these errors can lead to gait instability (Zhang et al., 2015) and therefore to falls. To safely classify thousands of daily steps in everyday use, a near-perfect classification performance is needed. Furthermore, the finite number of operation modes limits the user's ability to intuitively and spontaneously use a prosthesis in varying situations. Therefore, we consider state-dependent controllers not suitable for the volitional control system proposed in Section 3 and do not further discuss them.

#### 2.2.2 Reflex-driven muscle model based control

Reflexive, involuntary responses to stimuli are present in a large variety of human motor tasks and are believed to play a role in able-bodied gait (Kandel et al., 2012). Following preceding simulation studies (Geyer and Herr, 2010), some studies employed muscle and joint models driven by simulated reflexes instead of EMG signals. In this way, walking with an ankle prosthesis was proven possible, while enabling adaption to changes in slope (Eilenberg et al., 2010) and speed (Markowitz et al., 2011). Thatte et al. (2018) extended the concept to a knee and ankle prosthesis. Here, five able-bodied individuals using an adapter were able to walk with the prosthesis on level-ground. All three studies only implemented reflex control in stance phase and relied on predetermined trajectories during swing.

Like other CIC methods, this control strategy lacks volitional input from the user, and the presented implementations rely on state detection for gait. The reflex models used in the presented papers do not resemble actual biolocial control schemes (Markowitz et al., 2011), as reflexes in the human body are task-dependent and adapt to afferent neural signals from the brain (Kandel et al., 2012). Therefore, more complex reflexive networks with more detailed state distinctions are likely needed to enable different tasks like postural balance or sit-to-stand transitions.

### 2.3 Hybrid approaches

Some studies combine IEC and CIC elements in hybrid prosthesis control strategies. For stair ascent with a knee prosthesis, Hoover et al. (2013) modulated the stiffness and equilibrium point of an impedance controller via both EMG input and predefined values for stance and swing phases. Wang et al. (2013) used EMG to manipulate plantarflexion force during push-off with an otherwise intrinsic controller in an ankle prosthesis, which enabled level-ground walking. This was adapted to also support stair ambulation by Kannape and Herr (2014). Shu et al. (2022) applied a similar control approach with an offline optimization method to map EMG inputs to the virtual muscles.

Hybrid control approaches allow for some volitional adjustments to the assistance given by the device. Because they do not solely rely on volitional input, they feature a potential reduction in cognitive load compared to full IEC. On the contrary, the presented methods still carry the disadvantages of finite state controllers. They rely on the detection of gait phases and are limited to predetermined movements and activities.

## 3 Promising research directions

**Figure 2:**
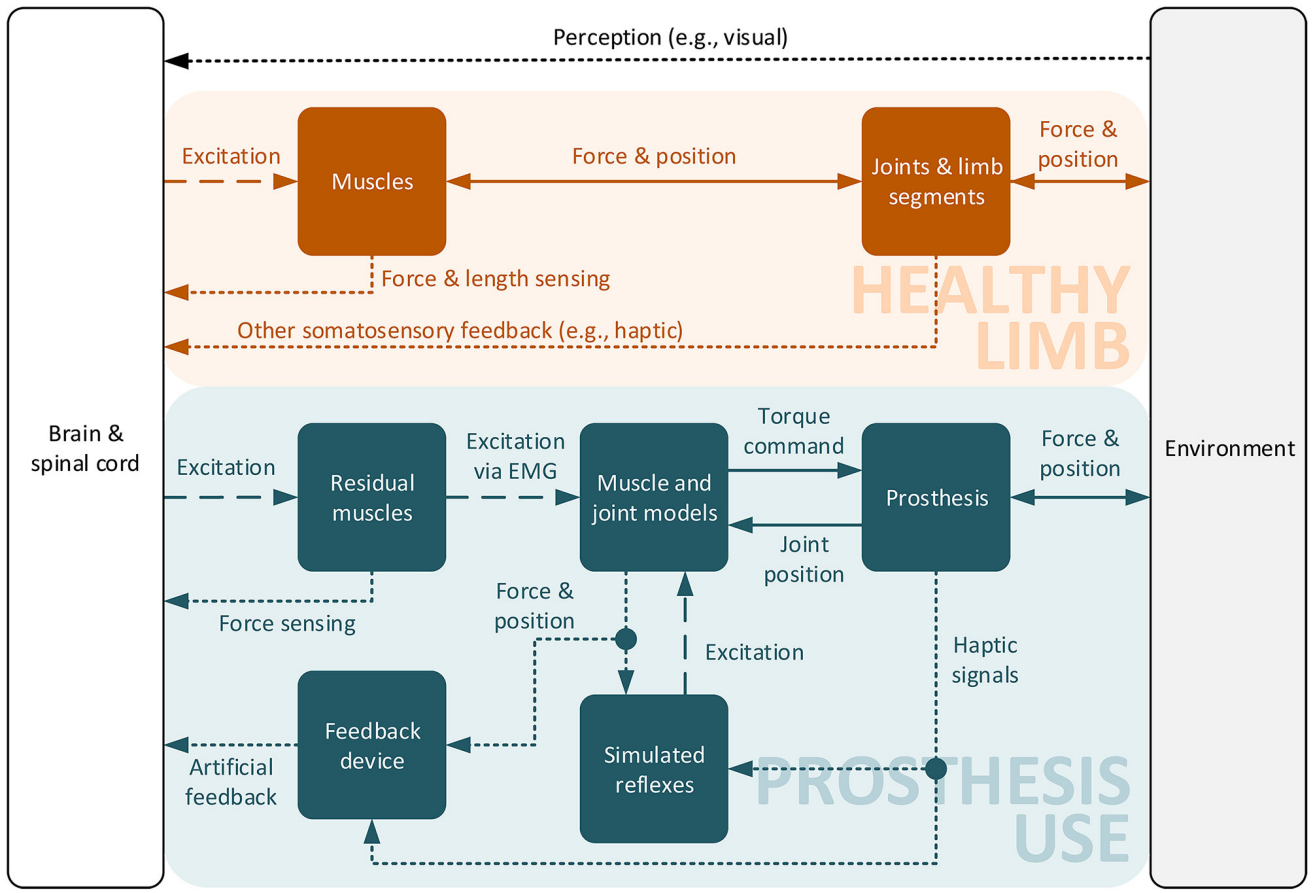
Diagram of control and excitation signals in the proposed control structure compared to a healthy limb. Elements and signals are orange for a healthy limb and blue in case of using a prosthesis. Black elements are present in both. Solid lines depict physical interaction or its virtual emulation. In the same way, dashed and dotted lines show efferent and afferent neural signals or their simulation.

Considering the previously analyzed control strategies, this section proposes future research directions to improve volitional lower limb prosthesis control. This suggestion includes parameterization approaches as well as the restoration of feedback and proprioception to prosthesis users. Figure 2 shows the proposed overall control structure in comparison with the motor control present in a healthy limb.

### 3.1 Combining EMG input and simulated reflexes

Human motor control signals are believed to consist of feed-forward and feedback components (Kandel et al., 2012). Markowitz et al. (2011) recognize this circumstance, but do not incorporate any user-controllable feed-forward signals in their control approach. Likewise, Shu et al. (2022) call reflexes essential for physiological gait and lament its absence in amputation musculature, but their device control relies on feed-forward signals alone. To our knowledge, the combination of muscle reflexes with feed-forward signals has only been tested in gait simulation, where it improved the model's robustness against perturbations compared to pure reflex control (Haeufle et al., 2018).

To facilitate compliant device behavior while limiting the cognitive demand on the user, we propose the development of a hybrid controller that combines EMG-based volitional input and simulated reflexes for the virtual muscle excitation in a model-based prosthesis control architecture. The structure of the proposed control approach is shown in Figure 2. Via EMG readings from residual muscles, the simulated muscles can be volitionally driven by the user, which subsequently command a desired torque to the prosthesis. Compared to the healthy limb, the afferent signals reporting muscle length (muscle spindles) and force (Golgi tendon organs) as well as cutaneous sensing to the spinal cord and brain (Kandel et al., 2012) are missing. This is substituted by incorporating virtual muscle states as well as sensory signals from the prosthesis hardware into simulated reflexes, which leads to an additional, non-volitional activation of the virtual muscles.

While this combined approach entails a high parameter count, it also allows for extensive individualization. Similar to the simulations conducted by Haeufle et al. (2018), the emphasis could be shifted between reflexes and volitional input by varying signal gains, depending on the wearer and their abilities to cognitively and physically handle the input responsibility. It is important to note that the studies mentioned here and in Section 2.2.2 mostly simulate monosynaptic stretch reflexes with a positive feedback of muscle force. This approach is self-reinforcing and would quickly lead to a maximum contraction of all muscles in a volitionally controllable prosthesis. Therefore, the reflex model needs to be adapted.

### 3.2 Restoration of feedback

The lack of haptic and proprioceptive sensing in prostheses limits the natural and intuitive movement dexterity of users. Supplementary feedback can improve immediate control performance and promote the learning of internal motor models (Sensinger and Dosen, 2020). Feedback of the device state to the user is suggested to facilitate embodiment, device acceptance and dexterity (Beckerle et al., 2019).

Providing position feedback in an EMG-controlled position tracking task can heavily improve performance. Canino and Fite (2016) demonstrated this in a knee prosthesis with both, static pressure and vibratory feedback. The presented studies examining prosthetic gait did not employ such feedback mechanisms, while some mention the necessity of visual feedback. A completely different approach to proprioception restoration is the agonist-antagonist myoneural interface, which aims to recreate natural co-dependencies by attaching residual muscles to their respective antagonist in the residual limb (Clites et al., 2018). In addition to improved volitional muscle activation, this also enables the introduction of perceived passive movement by electrically stimulating the muscles. This surgical construct has enabled great control performance for individuals with an amputation, including the presented hybrid approach by Shu et al. (2022).

Given the discovered benefits of prosthesis state feedback to the user, it should receive more attention in lower-limb prosthesis research. Since advanced and novel surgery procedures will likely not be available to or suitable for everyone, feedback mechanisms built into devices need to be investigated and developed further. Current solutions for artificial feedback do not match natural fidelity, while selecting the appropriate feedback signals remains a challenge (Seminara et al., 2023). As pictured by Beckerle et al. (2017), the form and extent of the provided artificial feedback should be thoroughly assessed to ensure actual improvements in a given task. To enable conscious reactions to changes in the artificial limb state, the structure given in Figure 2 contains a feedback device, which receives the same simulated and sensor feedback as the artificial reflexes. It is ceded to future research to design the feedback derived from these signals.

### 3.3 Human-in-the-loop optimization

Finding control parameters is a key challenge and choosing universal parameters for multiple users often does not appear suitable. The assistance required for a certain objective varies between individual users (Koller et al., 2015). People respond differently to active assistance and small changes can have large effects on energy expenditure (Voloshina and Collins, 2020). Thatte et al. (2018) observed that individual users prefer different gait characteristics and control parameter sets and called for methods to individualize prosthesis control.

Human-in-the-loop optimization (HILO) is a procedure of varying system parameters during user experiments to optimize for a given objective. It has been shown to successfully improve performance for exoskeletons and prostheses in a variety of tasks (Díaz et al., 2022) and offers the possibility to individualize prosthesis control (Voloshina and Collins, 2020). A common goal is the reduction of metabolic cost, which can be estimated from respiratory data (Zhang et al., 2017). An example for kinematic objectives is gait symmetry (Wen et al., 2020), which is especially interesting in the case of unilateral amputations. While HILO has been tested on a variety of control approaches for assistive devices, the combination with volitional control in lower-limb prostheses has yet to be realized.

We propose the use of HILO to determine control parameters that suit the individual needs of prosthesis users. Besides helping to manage large parameter spaces, HILO allows reseachers to pick different optimization objectives for different users or usecases. This can include desired gait kinetics, gait symmetry, metabolic cost, or user comfort and preference. For the last two, manual user input can be incorporated (Ingraham et al., 2020). Likewise, HILO allows to optimize for different tasks, like walking, stair-ascent and -descent or standing balance, depending on what is considered most important or most challenging by the user. To find initial parameters, anthropometric features and isometric torque and EMG measurements (Durandau et al., 2019) or predictive simulations of the human-machine interaction (Koelewijn and Selinger, 2022) can be used.

### 3.4 Consideration of model complexity

The studies on model-based EMG control mentioned in Section 2.1.2 all base their control on two simulated muscles in one degree of freedom (DoF) prostheses (plantar-/dorsiflexion for the ankle, flexion/extension for the knee). More elaborate models find use in CIC approaches (Markowitz et al., 2011; Thatte et al., 2018) and are being investigated for volitional control (Cimolato et al., 2020), but are not yet employed. Considering the example of an ankle prosthesis, active inversion/eversion was shown to reduce metabolic (Kim and Collins, 2017) and the lack of this DoF limits the adaption to uneven grounds. The use of only one plantarflexor muscle neglects the influence of the knee angle on the gastrocnemius length and hence the joint torque.

Challenging this common level of modeling depth could reveal potential benefits of more complex musculoskeletal models, though increase computational demands and parameter count. Therefore, the benefit of modeling certain biological anatomical features should be assessed and evaluated against the associated costs.

## 4 Conclusion

While active lower limb prostheses offer a multitude of potential benefits to users, their control remains challenging. In this perspective, we present arguments for the development of prostheses that are intuitively and volitionally controllable. Existing control approaches are summarized and evaluated regarding their suitability for that objective, laying out their advantages and disadvantages.

Overall, we propose the combination of EMG-based volitional control signals with simulated muscle reflexes in a model-based high level prosthesis control, aided by a feedback of the device's state to the user. We argue to strive for individualizing the assistance given by this control scheme to individual users and their respective needs. To this end and to tackle the potentially large number of model parameters influencing the control behavior, we argue for the use of HILO. We believe that this combination of methods will substantially advance the field of volitional control of lower limb prostheses and facilitate the development of intuitive to use leg prostheses in the future.

## Data availability statement

The original contributions presented in the study are included in the article/supplementary material, further inquiries can be directed to the corresponding author.

## Author contributions

MV: Conceptualization, Investigation, Visualization, Writing – original draft, Writing – review & editing. AK: Writing – review & editing. PB: Conceptualization, Supervision, Writing – review & editing.
